# Bone Augmentation of Peri-Implant Dehiscence Defects Using Multilaminated Small Intestinal Submucosa as a Barrier Membrane: An Experimental Study in Dogs

**DOI:** 10.1155/2019/8962730

**Published:** 2019-11-16

**Authors:** Siwen Wang, Weiyi Wu, Yuhua Liu, Xinzhi Wang, Lin Tang, Pengyue You, Jianmin Han, Bowen Li, Yi Zhang, Mei Wang

**Affiliations:** ^1^Department of Prosthodontics, Peking University School and Hospital of Stomatology, National Engineering Laboratory for Digital and Material Technology of Stomatology, Beijing, China; ^2^Department of Dental Materials, Peking University School and Hospital of Stomatology, National Engineering Laboratory for Digital and Material Technology of Stomatology, Beijing, China

## Abstract

**Objective:**

The aim of the study is to evaluate the effects of multilaminated small intestinal submucosa (mSIS) combined with bone substitute material to repair peri-implant defects during guided bone regeneration procedures.

**Methods:**

Twelve implants were placed in bilateral lower premolars of three beagle dogs, and a peri-implant buccal bone defect (3 mm width and 4 mm height) was created at each implant site. A total of 12 sites were filled with a particulate bone substitute material and then randomly divided into three treatment groups: covered by mSIS membrane (mSIS group), covered by collagen membrane (BG group), and no treatment (control group), each group of four sites. After 12 weeks of healing, all of the animals were euthanized and dissected blocks were obtained for micro-computed tomography (micro-CT) and histological analyses.

**Results:**

Micro-CT results revealed similar horizontal width of augmented tissue and new bone formation between mSIS and BG groups (*P* < 0.05). Histological analyses revealed that the differences in horizontal widths of newly formed bone and bone-to-implant contact between mSIS and BG groups were not significant (*P* > 0.05). All of these parameters were significantly different from those in the control group (*P* < 0.05).

**Conclusions:**

These findings confirmed that mSIS combined with the bone substitute material enhanced bone regeneration in peri-implant defects, in a manner similar to that of a collagen membrane.

## 1. Introduction

Insufficient alveolar bone volume after tooth extraction often leads to bone defects around the implant, which influences the long-term prognosis of the implantation treatment [1, 2]. The guided bone regeneration (GBR) technique, which combines different barrier membranes with the bone substitute material, has become the standard procedure in bone augmentation treatment of peri-implant dehiscence-type defects [3–5]. Among the bone substitute materials, deproteinized bovine bone mineral (DBBM) particles have been commonly used for its similar architecture to human bone trabecula and can support vascularization in early new bone formation. And bioabsorbable collagen membrane is the most frequently used barrier membrane in GBR treatments [6, 7].

The extracellular matrix (ECM), which retains collagen and various signaling molecules containing glycosaminoglycans, glycoproteins, and abundant growth factors [8], has been used in the GBR technique for the purpose of building a tissue engineering scaffold [9–12]. Porcine-derived small intestinal submucosa (SIS) is an acellular, naturally derived ECM material from the submucosal layer of porcine intestinal tissue [13, 14]. Previous *in vitro* studies have observed that the excellent biocompatibility of SIS is beneficial for adhesion, proliferation, migration, and osteogenic differentiation of cells which promote bone regeneration [15]. Moreover, SIS has demonstrated osteoinductive potential for use in the GBR procedure [16]. The multilaminated small intestinal submucosa (mSIS) membrane was used in this study, as it was shown to exhibit higher mechanical strength in a prior preclinical study [17]. The use of mSIS with enhanced mechanical strength, combined with the bone substitute material in the GBR procedure, significantly promoted new bone formation in bone defects [17], which suggests that the mSIS membrane may be useful as a GBR barrier membrane in clinical applications. Thus far, no studies have evaluated the effects of mSIS in animal models in closer proximity to clinical peri-implant defects.

Therefore, we established a mandibular peri-implant dehiscence defect model in beagle dogs, and the aim of the present study was to evaluate the effects of mSIS membrane combined with widely used bone substitute materials on bone augmentation of peri-implant buccal dehiscence defects.

## 2. Materials and Methods

### 2.1. Experimental Animals

Three healthy male beagle dogs with fully permanent dentition were included in this study (age, 18 months; weight, 14.5–17.5 kg). All animals were fed soft food throughout the study. This research protocol was approved by the Ethics Committee of Peking University (LA2017199).

### 2.2. Preparation of mSIS

The mSIS membrane was provided by Dasting Biological Technology Co., Ltd. The mSIS comprised eight layers of acellular monolayer porcine SIS, produced as a lyophilized stack. The manufacturing process is described in a previous paper [17].

### 2.3. Surgical Procedure 1

One experienced operator performed the whole surgery. The experimental animals were anesthetized by intravenous injection of 3% pentobarbital sodium at a dose of 1 mL/kg. After additional infiltration anesthesia was given at the surgical sites with articaine, the bilateral mandibular second, third, and fourth premolars (P2–P4) were extracted after being hemisected with fissure burs. A 5–0 nonabsorbable nylon suture was used to close the extraction site; the suture was removed after 10 days.

### 2.4. Surgical Procedure 2

**Figure 1:**
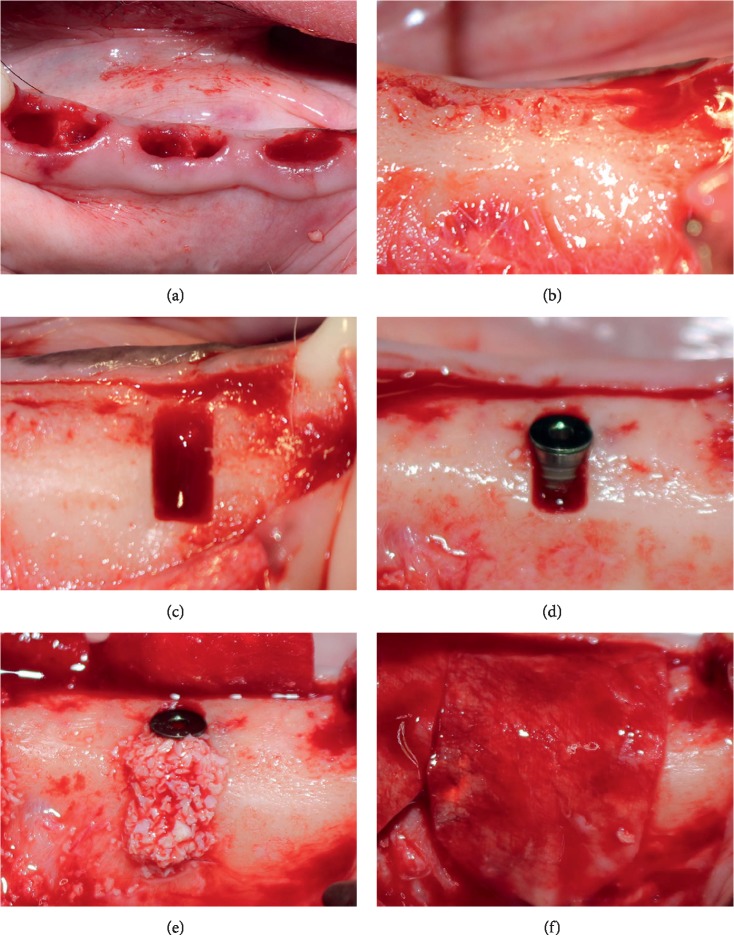
Surgical procedures. (a) The premolars (P2–4) were extracted. (b) After 12 weeks of healing, the alveolar bone was exposed. (c) Buccal defects were created (3 mm width and 4 mm height) and (d) implants were inserted. (e) In the control group, buccal defects were filled with bone substitute material, without covering membrane. (f) In the mSIS and BG groups, mSIS or collagen membrane was placed on each defect.

In order to simulate the implant placement in the edentulous alveolar ridge, we performed the second surgical procedure at 12 weeks of healing. The surgery was under general anesthesia similar to that of the first surgical procedure. A midcrest incision was made between the first premolar and the first molar on each side of the mandible, in addition to two vertical incisions through the mucogingival junction into the alveolar mucosa. After the mucoperiosteal flap was elevated and the alveolar bone exposed, the standard implant drilling procedures were prepared according to the manual of the Bego® system. The initial drill was applied first for directional orientation, and then, the 2.5 mm and 3.25 mm-diameter depth drills were used sequentially to the depth of 8.5 mm. Under copious irrigation with sterile saline, two standard dehiscence defects (a mesiodistal width of 3 mm and height of 4 mm, standardized using a periodontal probe) were created on each side of the mandible with turbine and fissure carbide bur. After creating the defects, the 3.25 mm × 8.5 mm titanium implants with cylindrical geometry (Bego®, Lincoln, RI, USA) was inserted on each defect, and the platform was placed at the same level as the alveolar crest (Figure 1).

**Figure 2:**
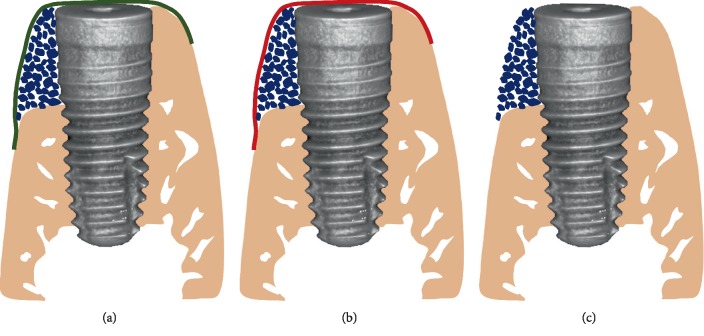
Schematic representations of treatments in all groups. (a) mSIS group: defects were filled with Bio-Oss and covered with mSIS. (b) BG group: defects were filled with Bio-Oss and covered with Bio-Gide. (c) Control group: defects were filled with Bio-Oss, without covering membrane.

Buccal defects were randomly allocated to three experimental groups: (1) mSIS group: defect was filled with the bone substitute material (Geistlich Bio-Oss® spongious bone substitute, granules 0.25–1 mm; Wolhusen, Switzerland) and covered with mSIS; (2) BG group: defect was filled with the bone substitute material and covered with a collagen membrane (Geistlich Bio-Gide®); and (3) control group: defect was filled with the bone substitute material, without covering membrane (Figure 2). Each buccal bone defects received about 0.06 g Bio-Oss® particles overbuilded after being soaked in normal saline for 5 min. Both membranes were trimmed to cover and extend 2-3 mm beyond the margin of the defect. The flap was repositioned and sutured with 5–0 nonabsorbable nylon after releasing the flap. All sutures were removed after 10 days.

### 2.5. Postoperative Management and Sacrifice

All animals received an intramuscular injection of penicillin G (800,000 U) immediately and every 24 h for 2 days after surgery. Plaque control was applied using 0.12% chlorhexidine for oral rinse, once per day for the first week after surgery and then twice weekly throughout the observation period. At 12 weeks after surgery, all animals were sacrificed with a fatal dose of pentobarbital sodium by intravenous injection. The mandibles including the surrounding alveolar bone and mucosa were block-resected and fixed in 10% neutral buffered formalin.

### 2.6. Micro-Computed Tomography Analyses

After fixation, the segments were scanned by using a micro-computed tomography (micro-CT) scanner (Siemens Inveon; Siemens, Munich, Germany) with an accelerating potential of 80 kV and a beam current of 500 *μ*A. The three-dimensional (3D) images were reconstructed, and the defect areas were marked with different colors. New bone volume (NBV) and new bone height (NBH) were measured within the area. The image orientation was adjusted based on the long axis of the implant. After the coronal view of the center of the implant was selected, we measured the horizontal width of the bone-augmented region at the implant shoulder level (HW_0), as well as at 2 mm (HW_2), 3 mm (HW_3), and 4 mm (HW_4) apical to the implant shoulder.

### 2.7. Histological Analyses

**Figure 3:**
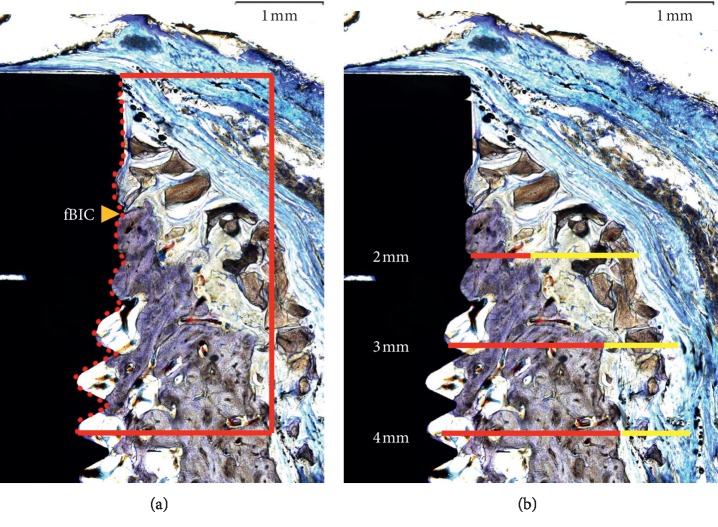
Schematic of histomorphometric analyses (magnification 40x). (a) Vertical linear and area measurements. B: bottom of the defect; fBIC: the first bone-to-implant contact. (b) Horizontal linear measurements. Yellow line: horizontal width of new bone; red line: horizontal width of augmented tissue.

After micro-CT analyses, the specimens were dehydrated through a gradient ethanol series and then infiltrated with methacrylate-based resin. The polymerized specimens were longitudinally sectioned at the center of each implant with the orientation along the buccal and lingual cross sections using the Exakt diamond cutting system (300 CP Exakt Advanced Technologies GmbH, Norderstedt, Germany) and then were attached to slides using the adhesive press system. The sections were finally ground to a thickness of 30–50 *μ*m. All slides were stained with toluidine blue. Then, a light microscope (CKX-41; Olympus, Tokyo, Japan) with a CCD camera was used to observe and capture images. The following landmarks were identified in the stained sections: bottom of the bone defect (B) and the first bone-to-implant contact (fBIC). The following parameters were analyzed (Figure 3):

- Percentage of new bone area within augmented area (NBA; %)
- Percentage of remaining bone substitute area within augmented area (RBS; %)
- Amount of bone-to-implant contact from B to the implant shoulder at magnification of 100x (BIC; %)
- Distance from B to fBIC (B-fBIC)
- Horizontal width of bone-augmented area at the implant shoulder level (ATT_0), as well as at 2 mm (ATT_2), 3 mm (ATT_3), and 4 mm (ATT_4) apical to the implant shoulder
- Horizontal width of new bone at the implant shoulder level (NBT_0), as well as at 2 mm (NBT_2), 3 mm (NBT_3), and 4 mm (NBT_4) apical to the implant shoulder

### 2.8. Statistical Analyses

The experimental data are expressed as means ± standard deviations. Statistical analyses were conducted using SPSS software (ver. 20.0; SPSS Inc., Chicago IL, USA). One-way analysis of variance was performed, and the LSD post hoc test was conducted. A *P* value <0.05 was considered significant.

## 3. Results

### 3.1. Clinical Findings

All animals survived to the end of the study and were in good condition; all 12 implant sites healed without complications. No implant failures or membrane exposures were observed.

### 3.2. Micro-CT Analyses

**Figure 4:**
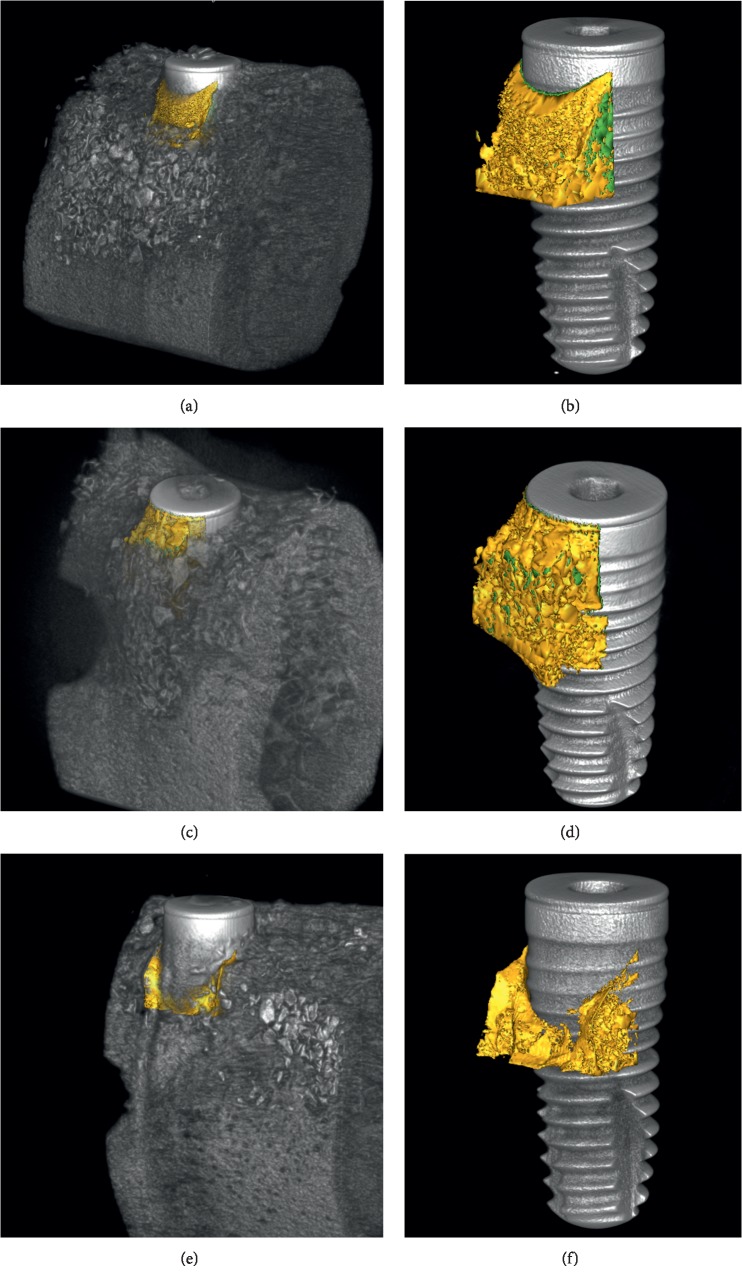
Three-dimensional micro-CT reconstructed images. (a, c, e) Block sections in the mSIS, BG, and control groups. (b, d, f) Images without old bone in the mSIS, BG, and control groups.

The bone substitute material was located in the buccal region of the implant. A large amount of residual bone substitute material was found in both BG and mSIS groups, a portion of which was displaced slightly to the apical region. In the control group, only a small amount of residual bone substitute material was detected in the apical region of the implant (Figure 4).

**Figure 5:**
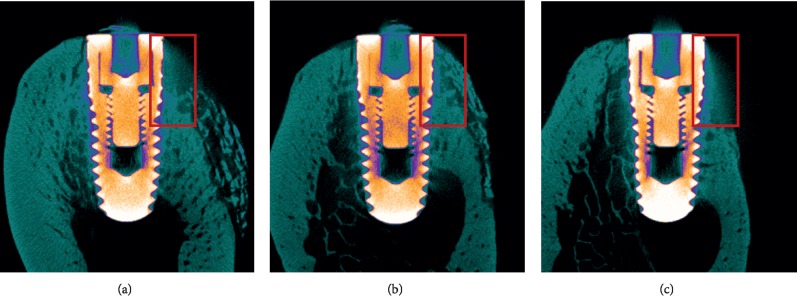
Buccolingual section images show new bone and bone substitute materials. (a) mSIS group. (b) BG group. (c) Control group.

The buccal and lingual cross-sectional image of the BG and mSIS groups showed obvious new bone formation in the defect area, and the boundary with the old bone was indistinguishable. A large number of bone substitute material particles formed an arc shape from the implant abutment to the defect bottom in the augmented area. Some bone substitute material was scattered in the augmented area. Only a small amount of new bone formation was observed in the augmented area of the control group (Figure 5).

**Figure 6:**
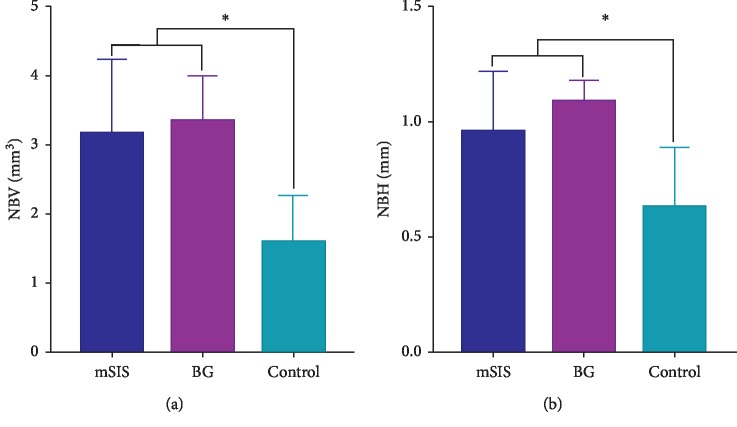
Quantification graph representing (a) NBV and (b) NBH. ^*∗*^Significantly different (*P* < 0.05).

The micro-CT measurements are summarized in Tables 1 and 2, as well as Figure 6. The mean NBV values of the mSIS and BG groups were 3.15–3.35 mm^3^, and the mean NBH values of the mSIS and BG groups were 0.96–1.09 mm; these were significantly higher than the values in the control group (*P* < 0.05), while no statistical differences were detected between the mSIS group and the BG group (*P* > 0.05).

At the level of the implant shoulder, the horizontal width of the bone-augmented region and the width of the new bone were 0.00 mm in each group. At the levels of 2 mm and 3 mm apical to the shoulder, the widths of bone-augmented tissue in the mSIS and BG groups were larger than those in the control group. At 4 mm apical to the shoulder, the widths of bone-augmented tissue in the mSIS and BG groups were significantly larger than that in the control group (*P* < 0.05); the difference between the mSIS and BG groups was not statistically significant (*P* > 0.05).

### 3.3. Histological Findings

**Figure 7:**
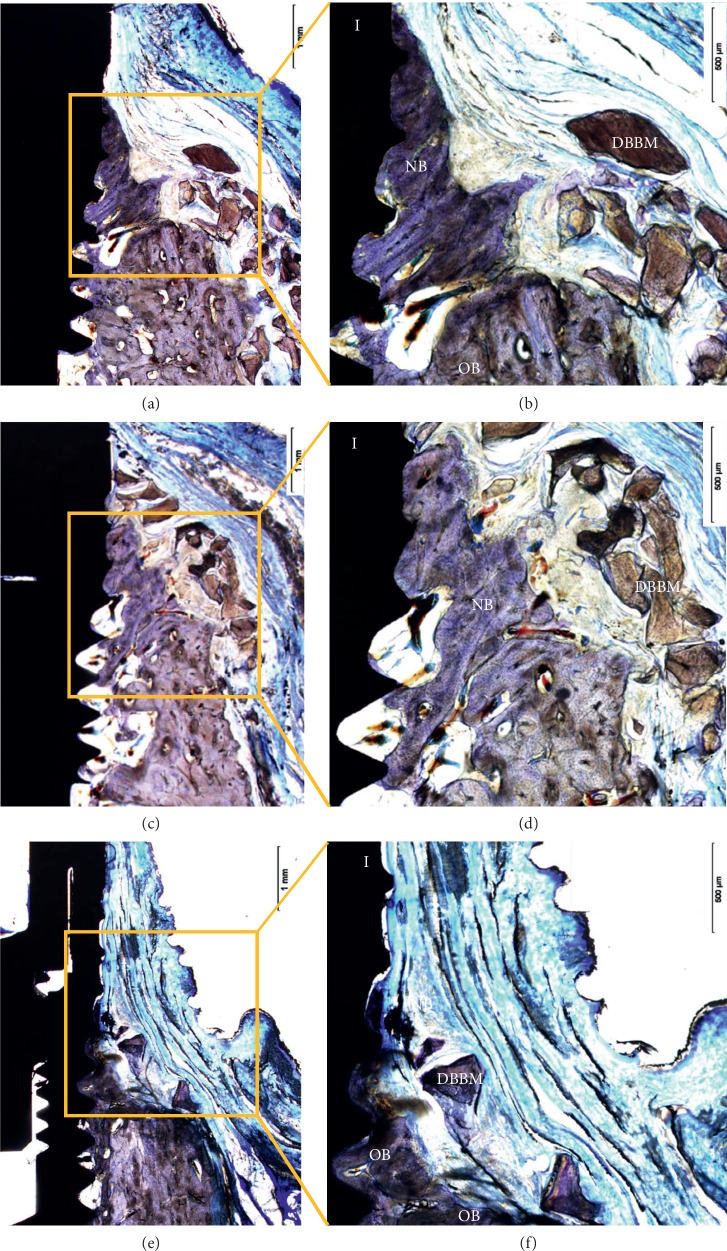
Histological sections of the mSIS (a, b), BG (c, d), and control groups (e, f). NB, new bone; OB, old bone; DBBM, bone substitute material; I, implant (magnification 12.5x (a c, e) and 40x (b d, f)).

A large amount of new bone tissue was observed in the augmented area in the mSIS (Figures 7(a) and 7(b)) and BG groups (Figures 7(c) and 7(d)), and the staining color was deeper than that of old bone. Part of the bone substitute material in the augmented area was embedded in new bone, the boundary of which was unclear. Some bone substitute material formed the outer contour of the augmented area. Part of the bone substitute material was displaced during the healing process and was located on the buccal and apical sides of the bone-augmented area, surrounded by connective tissue; a small amount of new bone tissue remained at the edge of the particles.

Only a small amount of new bone was observed in the control group, and residual bone substitute material was nearly absent (Figures 7(e) and 7(f)).

### 3.4. Histomorphometric Analyses

**Figure 8:**
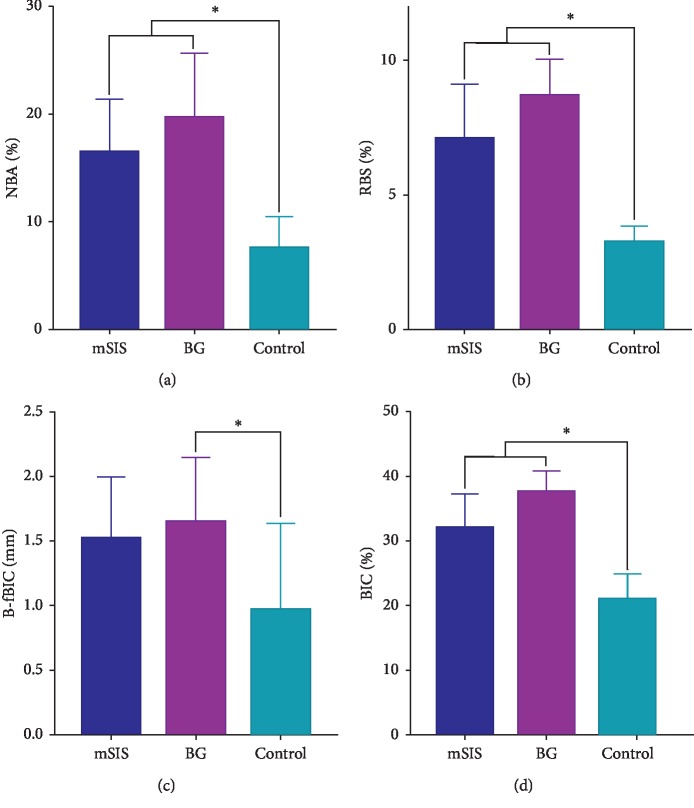
Quantification graph representing (a) NBA, (b) RBS, (c) B-fBIC, and (d) BIC. ^*∗*^Significantly different (*P* < 0.05).

Histomorphometric measurements are summarized in Tables 3–5 and Figure 8. The area ratios of remaining bone substitute materials (RBS (%)) and BIC (%) in the mSIS and BG groups were significantly higher than those in the control group (*P* < 0.05), while the differences between mSIS and BG groups were not significant (*P* > 0.05). The mean B-fBIC value in the BG group was significantly higher than that in the control group (*P* < 0.05), whereas the mean B-fBIC value in the mSIS group tended to be numerically greater than that in the control group (*P* > 0.05).

At the level of the implant shoulder, the widths of the bone-augmented area and of the new bone were 0.00 mm in each group. At 2 mm and 3 mm below the abutment, the widths of the bone increment and of the new bone in the mSIS and BG groups were larger than those in the control group. At 4 mm below the abutment, the widths of bone-augmented area in the BG group was significantly larger than that in the control group (*P* < 0.05), whereas the difference between the mSIS and BG groups was not statistically significant (*P* > 0.05). The new bone widths in the mSIS and BG groups were significantly larger than that in the control group (*P* < 0.05); the difference between the mSIS and BG groups was not statistically significant (*P* > 0.05).

## 4. Discussion

Peri-implant buccal defects are among the most common problems encountered in the clinic. Peri-implant defects often occur after implant placement when the width of the alveolar bone is insufficient. The use of the GBR technique to place the bone substitute material combined with the collagen membrane promotes new bone formation in peri-implant defects [18, 19]. Because of the high degree of similarity between the jaw bones of humans and dogs with regard to bone density and structure [20, 21], dogs are often used to construct models of canine peri-implant defects to evaluate the stability of bone-augmented areas following treatment with a GBR membrane. To the best of our knowledge, this is the first study to apply the mSIS membrane for treatment of this type of peri-implant dehiscence defect. As in previous studies that have used a similar protocol in a canine peri-implant defect model [22, 23], new bone formation was consistent in the mSIS and BG groups in our study, indicating that the bone defect model we established is a valid experimental model.

As an ECM material, the mSIS membrane used in this study has the advantages of collagen membrane and retains various growth factors after a series of manufacturing processes; these include fibroblast growth factor (FGF), vascular endothelial growth factor (VEGF), and transforming growth factor (TGF). Previous studies have shown that the SIS membrane is beneficial with regard to new bone formation and maturation in GBR treatment [16]; it can recruit different cells that express factors related to bone regeneration and bone remodeling such as bone morphogenetic protein-2 and TGF-*β*. In a rat cranial defect model, significant new bone formation can be observed in defect areas covered by the SIS membrane, particularly in areas close to the membrane [16]. Similar results have been achieved using mSIS combined with the bone substitute material in mandibular bone defects in a rabbit model [17]. Here, we compared the mSIS membrane with widely used collagen membranes for GBR in beagle implant models utilizing clinical methods.

New bone volumes of the mSIS and BG groups were 3.15 mm^3^ and 3.35 mm^3^, respectively, both significantly higher than in the control group. In 3D reconstruction images, we observed more new bone volume near the margin of the defect, which gradually decreased as it approached the center. To characterize new bone formation in the central part of the defect, histological analyses of buccal and lingual cross sections were performed. The new bone area ratio of the mSIS group was 16.43%, similar to that of the BG group (19.66%); both ratios were significantly higher than those of the control group. This is consistent with studies of Won et al. [23] and Jung et al. [24] and demonstrates that the mSIS and Bio-Gide membrane enhanced new bone formation in the center of the defect, compared to the control group (without a covering membrane).

Implant stability requires good osseointegration. In this study, the bone-to-implant contacts of the defect area in the mSIS and BG groups were 33.30% and 39.19%, respectively; these values were not significantly different. A previous study that used a similar bone defect model obtained 40.22% bone-to-implant contact at 8 weeks after bone augmentation [22]. Another study reported a 36.25% bone-to-implant contact at 16 weeks after application of the collagen membrane combined with the bone graft material [19]; our results are consistent with those findings. This indicates that new bone that forms in defect areas covered by an mSIS membrane can achieve osseointegration similar to that achieved with the Bio-Gide membrane.

Maintaining space stability of the bone-augmented area is critical for bone regeneration in the GBR procedure. Although the bone graft material was placed in each group, bone regeneration results in the mSIS and BG groups were significantly better than in the control group. The 3D reconstruction images showed that there was considerable retention of bone substitute materials in the mSIS and BG groups in the bone-augmented region, whereas retention was minimal in the control group. These results are similar to those of a previous study in which a dehiscence-type defect model was used but different from the results using a saddle-type defect, which exhibited a wider margin [25]; this discrepancy may be related to differences in the types of bone defects. A narrower bone margin in the dehiscence-type defect provided less support for the bone substitute material. Both wound closure and pressure from the overlapping soft tissue may have caused apical displacement of the bone substitute material [18, 26–28]. Therefore, a barrier membrane with greater mechanical strength and stiffness is more favorable for maintenance of the augmented area and retention of the bone substitute material. The mSIS membrane used in this study comprised eight layers of monolayer mucosa and had superior mechanical properties relative to the collagen membrane when wet. The results of histological quantitative analyses showed that the horizontal widths of new bone in the mSIS group at 2, 3, and 4 mm apical to the implant shoulder were higher than those in the control group, indicating that the mSIS membrane might positively affect the retention of bone substitute materials in the early healing stage; this could aid the maintenance of a stable space for new bone formation.

The mSIS membrane maintained the stability of the bone-augmented region in peri-implant buccal bone defects, similar to the Bio-Gide membrane, which supports the clinical applicability of the mSIS membrane. However, because of the limited number of experimental animals used in the study, we could not obtain additional supportive data. In addition, there were numerical differences between the results of the mSIS and BG groups. The increased number of layers could improve the mechanical strength of the material, but this led to increased stiffness, which was unfavorable for flexibility during the operation. During the surgical procedure, the ability of the mSIS to fit the contour of the bone defect was slightly inferior to that of the collagen membrane, which may have resulted in the displacement of more bone substitute materials in the mSIS group. Therefore, the ideal number of layers of mSIS membrane that provides both appropriate stiffness and flexibility for clinical manageability must be further investigated in subsequent studies.

## 5. Conclusion

Based on micro-CT and histomorphometric analyses, mSIS combined with a bone substitute material achieved new bone regeneration similar to that of a collagen membrane in a peri-implant dehiscence-type defect; the outcomes in both experimental groups were significantly better than those in the control group.

## Figures and Tables

**Table 1:** Micro-CT quantitative analyses in the area of interest (mean ± standard deviation).

	mSIS group	BG group	Control group
NBV (mm^3^)	3.15 ± 1.08^a^	3.35 ± 0.65^a^	1.60 ± 0.67^b^
NBH (mm)	0.96 ± 0.26^a^	1.09 ± 0.09^a^	0.63 ± 0.26^b^

NBV, new bone volume; NBH, new bone height; different letters indicate significant differences between groups in the same condition (*P* < 0.05).

**Table 2:** Micro-CT quantitative analysis of the horizontal width of bone augmentation area (mean ± standard deviation).

	mSIS group	BG group	Control group
HW_0 (mm)	0.00 ± 0.00	0.00 ± 0.00	0.00 ± 0.00
HW_2 (mm)	0.33 ± 0.20	0.40 ± 0.34	0.19 ± 0.14
HW_3 (mm)	1.03 ± 0.22	1.34 ± 0.44	0.79 ± 0.37
HW_4 (mm)	1.86 ± 0.18^a^	2.29 ± 0.17^a^	1.19 ± 0.38^b^

Different letters indicate significant differences between groups in the same condition (*P* < 0.05).

**Table 3:** Histometric analysis in the area of interest (mean ± standard deviation).

	mSIS group	BG group	Control group
NBA (%)	16.43 ± 4.96a	19.66 ± 6.02a	7.56 ± 2.91b
RBS (%)	7.09 ± 2.03a	8.68 ± 1.36a	3.27 ± 0.58b
B-fBIC (mm)	1.52 ± 0.48a, b	1.65 ± 0.50a	0.97 ± 0.67b
BIC (%)	33.30 ± 5.56a	39.19 ± 3.41a	21.92 ± 4.14b

NBA, new bone area; RBS, remaining bone substitute; B-fBIC, distance from the first bone-implant contact to the bottom of the defect; BIC, bone-to-implant contact; different letters indicate significant differences between groups in the same condition (*P* < 0.05).

**Table 4:** Histometric analysis of the horizontal width of bone augmentation area (mean ± standard deviation).

	mSIS group	BG group	Control group
ATT_0 (mm)	0.00 ± 0.00	0.00 ± 0.00	0.00 ± 0.00
ATT_2 (mm)	0.36 ± 0.37	0.54 ± 0.55	0.15 ± 0.03
ATT_3 (mm)	1.13 ± 0.27	1.25 ± 0.53	0.59 ± 0.16
ATT_4 (mm)	1.85 ± 0.38a, b	1.91 ± 0.59a	1.16 ± 0.34b

Different letters indicate significant differences between groups in the same condition (*P* < 0.05).

**Table 5:** Histometric analysis of the horizontal width of new formation bone (mean ± standard deviation).

	mSIS group	BG group	Control group
NBT_0 (mm)	0.00 ± 0.00	0.00 ± 0.00	0.00 ± 0.00
NBT_2 (mm)	0.11 ± 0.12	0.15 ± 018	0.15 ± 0.03
NBT_3 (mm)	0.76 ± 0.33	1.03 ± 0.72	0.37 ± 0.16
NBT_4 (mm)	1.42 ± 0.35a	1.52 ± 0.42a	0.76 ± 0.26b

Different letters indicate significant differences between groups in the same condition (*P* < 0.05).

## Data Availability

The data used to support the findings of this study are available from the corresponding author upon request.
